# Built environment stakeholders’ experiences of implementing healthy urban development: an exploratory study

**DOI:** 10.1080/23748834.2021.1876376

**Published:** 2021-01-22

**Authors:** Helen Pineo, Gemma Moore

**Affiliations:** Institute for Environmental Design and Engineering, Bartlett School of Environment, Energy and Resources, University College London, London, UK

**Keywords:** Design, development, health and wellbeing, planning

## Abstract

Healthy urban development, in the form of buildings and infrastructure, is necessary to reduce disease and injury internationally. The urban development process is complex, characterised by a plurality of actors, decisions, delays, and competing priorities that affect the integration of health and wellbeing. Despite clear shifts in the built environment sector towards considering health, there is a lack of research about how the principles of healthy design are put into practice in development projects. We explored this topic via semi-structured interviews with 31 built environment and public health professionals involved in such projects in Australia, China, England, the Netherlands, Sweden and the United States. We used thematic analysis and three themes emerged from our hybrid deductive and inductive approach, encompassing challenges and potential solutions for integrating health in development. Managing risk, responsibility and economic constraints were paramount to persuade developers to adopt healthy design measures. Participants could push business-as-usual practices towards healthy urbanism by showing economic benefits or piloting new approaches. Finally, participants had contrasting views on whether increasing professional knowledge is required, with several arguing that financial barriers are more problematic than knowledge gaps. This exploratory study contributes insights into an under-research topic and outlines priorities for further investigation.

## Introduction

Changes to urban environments in the form of buildings, infrastructure and public spaces affect health and wellbeing. This fact is widely recognised in academic literature, yet relatively new to the educational curricula and professional practice of those who make decisions about urban development (e.g. planners, urban designers, developers, architects, surveyors and others). Many international and national policy agendas promote healthy urban development principles and professional bodies provide guidance and case studies to inspire good practice. Yet, there is often a question about how far the aspirations of policy and guidance can be connected to the reality of implementing change on the ground. Applying healthy development principles is perceived to be difficult by professionals for a wide range of reasons including unsupportive regulatory systems, lack of economic viability, and the complexity of the development process (McGreevy *et al*. 2019, Carmichael *et al*. 2020).

Scholars, governments and professional organisations have produced frameworks and design guidance for healthy urban environments that aim to influence planning, design and construction (e.g. UN-Habitat and World Health Organization 2020; Urban Land Institute 2015, WHO 2018, Clements-Croome *et al*. 2019). Relevant planning and design strategies include those which are: spatial and infrastructural (e.g. walkable neighbourhoods, networks of green infrastructure, integrated transport systems), technological (e.g. air quality sensors or low toxicity building materials), architectural (e.g. adequate space and thermal comfort) and social (e.g. accessible open space and affordable housing). Pineo (2020) reviewed 15 healthy urban design and planning guidance documents and argued that many perpetuated a narrow model of health, one which emphasises supporting ‘healthy lifestyle choices’ (e.g. physical activity and diet) rather than recognising structural barriers to health and the urgent risks of environmental degradation. The review indicates a gap between the social and environmental justice imperatives of healthy urbanism and the existing professional guidance.

Creating healthy developments is challenging in part because the development process is highly complex and no single actor or institution is in control; they are part of a system responding to and managing the effects of each others’ activities over time (Rydin *et al*. 2012). Urban development is ‘the process of physically producing the built environment, by bringing together multiple actors from construction companies to development financiers to local planners and others’ (Rydin 2010, p. 15). Development may be large or small in scale, including major urban redevelopment projects and incremental improvements to existing buildings (ibid). Urban development processes are characterised by a plurality of decisions, stakeholders and competing priorities that create both challenges and opportunities for the integration of health and wellbeing. Urban designers and planners, land owners, developers, investors, communities and others have key roles to play in creating healthy places. Scholars have highlighted the importance of building professional relationships across sectors (and community groups) to support collaboration and knowledge sharing for healthy development. This is particularly important since healthy planning and design are not typically covered in university curricula for built environment professionals (Pilkington *et al*. 2013, Marsh *et al*. 2020).

Looking upstream in the development process, there has been considerable research on the process of integrating health into urban planning policy, with some research on implementation. Challenges for healthy planning policy include: a requirement for more localised and simplified evidence about environmental health impacts and associated economic arguments, lack of a shared understanding of ‘health’ across policy actors, ‘silo’ working across relevant sectors, conflicting goals across tiers of government and under-resourcing of delivery mechanisms (Carmichael *et al*. 2012, 2019, Fazli *et al*. 2017, Lowe *et al*. 2018, Ige-Elegbede *et al*. 2020, Pineo *et al*. 2020).

Implementation of healthy places is hindered by the perception that healthy places are more expensive to design, build and maintain. This has been identified as a key barrier across markets and different types of development (e.g. residential, mixed use, office) (Carmichael *et al*. 2012, Chang 2018, Design Council 2018). The costs and benefits of achieving healthy development are distributed across a wide range of actors, with differences in who pays and who benefits, making it difficult to easily demonstrate the ‘business case’ (Pineo and Rydin 2018). In many countries, this financial challenge is related to a reliance on private sector developers to deliver healthy places within the margins that can be reasonably expected from such investments (Rydin, 2013). Developers may argue that policy requirements tied to permission to build (including sustainability standards, impact fees/contributions and affordable housing) reduce their ability to meet additional healthy design objectives. This challenge has been raised by Carmichael *et al*. (2020) as they argue that ‘the existence of sub-standard housing in England can be seen as a market failure’ that is driven by ‘the legitimate use of viability assessment findings by developers to reduce the number of affordable homes, quality of the design, or size of the homes they are required to build’ (p.2). Scholars and professional bodies have worked with developers and other actors to quantify the financial value created through healthy placemaking to counteract the viability challenge (Kramer *et al*. 2014, Chang 2018, Carmona 2019).

Whether driven by an urge to bolster an unsupportive policy landscape or capitalise on a new market, healthy building rating tools have proliferated in recent years. Voluntary standards and assessment tools such as WELL, Fitwel and RESET are influencing development projects internationally (McArthur and Powell 2020) and creating a new mark of ‘value’ for buildings that promote health and wellbeing (Pineo and Rydin 2018). Sustainability rating tools have been credited as supporting occupant health (Colton *et al*. 2014, 2015, MacNaughton *et al*. 2017). However, they have also been criticised for being too costly, constraining innovation and failing to integrate local context (including social, environmental, and economic factors) into a one-size-fits-all approach (Ding 2008, Retzlaff 2009, Boyle *et al*. 2018). There is a lack of literature on healthy building rating tools, including studies of their application and measurable health impacts after construction. In a paper linked to our study, Callway *et al*. (2020) consider the role of such rating tools in the negotiation of health objectives in development processes.

Despite a clear international shift in the development sector towards healthy design and building, there is a lack of research about how healthy development policies are implemented in practice. There is a pressing need to understand the ‘jump’ from theory (i.e. the principles of healthy urbanism) to built environment professionals’ practice. A theory-practice gap has been identified and explored in other fields, including healthcare, where practitioners struggle with implementing research or theoretical knowledge in real-world settings (Nilsen 2015, Greenway *et al*. 2019). Within practice it can be challenging to apply principles and theories to specific contexts or situations. Furthermore, there can be discrepancies between what professionals’ claim underlie their practices and what implicit understandings and values are unknowingly embedded in their work. This study aims to explore how the principles of healthy urbanism are put into practice through investigating built environment professionals’ experiences of implementing health in new urban developments.

This exploratory study is part of a wider collaborative research project with Guy’s and St Thomas’ Charity on integrating health into new development. In this study we aim to provide insights into the healthy development process from the perspectives of experienced professionals working in international contexts, in Australia, China, England, the Netherlands, Sweden, and the USA. We explore the following questions through semi-structured interviews:

1) How is healthy urban development conceptualised and applied in practice by built environment professionals?
2) Which factors influence or drive the implementation of health in new urban development?
3) What are the opportunities and barriers for integrating health into new urban development?

Through exploring practitioners’ experiences, this scoping study contributes to knowledge of how healthy placemaking can be successfully delivered.

The paper is structured as follows: the next section briefly outlines the policy and practice context for healthy urbanism in the study’s geographic areas, followed by a description of our methodological approach. The findings begin with an overview of participants’ perceptions on the drivers of healthy development followed by descriptions of the important role of developers. We then describe the three themes that emerged from the thematic analysis: managing risk, responsibility and economic constraints; pushing business-as-usual practices; and building knowledge and capacity. The discussion section considers our findings in the context of wider literature and considers the strengths and limitations of the study. Our conclusions outline priority areas for further research.

### Drivers and status of healthy development internationally

Healthy urbanism is influenced by the agendas of international organisations, which are diversely interpreted and applied according to local contexts and priorities. The World Health Organization (WHO) has supported healthy built environment efforts, particularly through its Healthy Cities movement, since the 1980s (Hancock and Duhl 1986). The social and environmental determinants of health have been integrated with wider sustainable development priorities through the work of UN Habitat and others, exemplified by the Sustainable Development Goals’ explicit links between health and the environment (UN General Assembly 2015). As argued by Pineo (2020), an important driver for healthy property development relates to the framing of health and sustainability as overlapping goals by industry bodies such as the World Green Building Council (WGBC 2013, 2014, 2016) and Urban Land Institute (Kramer *et al*. 2014, ULI 2015, Hammerschmidt *et al*. 2016). This section briefly describes the policy and practice context for healthy urbanism in countries explored within this study.

#### Australia

The healthy built environment agenda in Australia has progressed through academic and government activities promoting ‘liveability’ and healthy planning, particularly in the more populated states of New South Wales and Victoria. Planners have benefited from guidance and assessment tools (Lowe *et al*. 2015, 2018, Paine and Thompson 2017, Kent and Thompson 2019). Similar to the US and Europe, issues of health equity are recognised in the Australian healthy planning literature. McGreevey *et al*. (2019) evaluated two planning processes in Adelaide and found that ‘liveability’ policies (i.e. health-promoting) could result in further investment in ‘image enhancing parts of the city, not to areas of social or locational disadvantage’ (p.7), further exacerbating existing inequalities. Pineo *et al*. (2020) found that implementation of healthy planning policies in new development near Sydney and Melbourne was heavily constrained by economic factors and the requirement for new housing.

#### China

The Healthy China 2030 strategy (Central Committee of Chinese Communist Party and State Council 2016) has been a significant driver for intersectoral action for health in China, including the development of healthy buildings (Wang *et al*. 2020). In response to the government’s health agenda and influence from the WELL standard, the Chinese Academy of Building Science published the Assessment Standard for Healthy Building (T/ASC02-2016) in January 2017, which defines healthy buildings as those ‘that can provide healthier environment, facilities and services, promote users’ physical and mental health, and achieve the improvement of health performance, on the basis of fundamental functions’ (ibid). There has been considerable adoption of the standard, yet some scholars do not believe it encompasses the right priorities for healthy urban development in China. Hu (2020) argues that the focus of healthy urban design should be through the regeneration of existing places, in the context of demand for more leisure and community spaces, design for all ages, and healthy indoor and outdoor environments. However, there is a lack of theory, standard, technique, and practice of healthy regeneration in China (ibid). Hu (2020) and Meng (2017) state that the key priorities for the Chinese healthy building agenda going forward are developing a more detailed standard system to guide design and construction, evaluating performance, and establishing industry networks/communication platforms.

#### England

The English planning system promotes health in its strategic National Planning Policy Framework, yet scholars and practitioners feel that this requirement has relatively low weight in decision-making compared to economic and housing development goals (McKinnon *et al*. 2020, Carmichael *et al*. 2020). A recent initiative by the National Health Service highlighted the links between development and health through ten Healthy New Town demonstrator sites (NHS England 2019). English practitioners often see healthy placemaking as being in competition with other development objectives and too costly to implement (Design Council 2018), yet there are active practitioner networks and interest among developers highlighted by the recent work of the Town and Country Planning Association (Chang 2018). The TCPA brought together housing developers, public health teams and others to explore how consensus and action regarding high-quality healthy development could be achieved. Key findings included the need to fix fundamental ‘flaws’ with the housing market, increasing the quality and availability of affordable homes, engaging early in the development process, identifying incentives to engage more developers, finding ways to share risks and rewards, improving the ‘commercial case’ and increasing the use of health evidence (ibid).

#### Sweden and the Netherlands

Cities in Sweden and the Netherlands are often cited as examples of sustainable and healthy urban form, particularly with regards to cycling infrastructure (Creutzig, Mühlhoff, and Römer 2012, Fishman *et al*. 2015). Recently, scholars have investigated the health impacts of urban regeneration in the Netherlands, yet the focus has been predominately on socioeconomic over environmental improvements (Droomers *et al*. 2014, 2016, Ruijsbroek *et al*. 2017). The European WHO Healthy Cities programme widely promoted healthy urban planning. Their work has increased: cross-sectoral health promotion activities; application of health impact assessments; and leadership supporting participatory governance, health equity and healthy ‘lifestyle programmes’ (de Leeuw *et al*. 2014, Grant 2015). Sweden and the Netherlands have policy initiatives and Healthy Cities networks (although the Dutch network is not accredited) encouraging healthy placemaking.

#### United States

The United States has been a leader in healthy placemaking research and practice, resulting in significant international influence (see Dannenberg *et al*. 2011, Galea *et al*. 2019). In recent years, professional bodies and their local chapters have published guidance, case studies and policy initiatives (e.g. Washington American Planning Association Game Changing Initiative Health and Planning Working Group 2016). This knowledge-building activity has coincided with the development of new healthy building standards (the WELL Building Standard, Living Building Challenge and Fitwel) that have subsequently influenced international practice. A recent review of building standards reports that WELL and Fitwel have been applied in 51 and 36 countries, respectively (McArthur and Powell 2020). These standards have ridden a wave of interest in health, comfort, well-being, and productivity in buildings across practitioners and scholars, and they have benefitted from explicit links to the green building agenda (Allen *et al*. 2015, Cedeño-Laurent *et al*. 2018, Allen and Macomber 2020). Recent research seeks to unpick the health impact of urban regeneration, with significant focus on structural inequities linked to the environment (see Schnake-Mahl *et al*. 2020).

In summary, the countries covered in this study have explicit drivers for healthy urban development. There is variation in their respective areas of focus and the availability of studies about policy implementation. This study focuses on professionals’ experiences of how policy is used to leverage health through projects across these regions, ranging from single buildings to large-scale developments.

## Methodology

We aim to understand the integration of health into new development through an interpretive exploration of professionals’ experiences and perceptions. Through adopting a qualitative, interpretative form of inquiry this study aimed to, as phrased by Schwandt (1994, p. 118), ‘elucidate the process of meaning construction and clarify what and how meanings are embodied in the language and actions of social actors’. We briefly report our methods for data collection and analysis below, with further detail in the supplementary material.

### Semi-structured interviews

We conducted semi-structured interviews with 31 professionals. We met 17 participants in Beijing, China (May 2019); London, England (June/July 2019); Seattle, USA (August 2019) and spoke with an additional 14 via Skype (June 2019 to February 2020 covering participants in Australia, England, Sweden and the Netherlands). We selected the geographic regions of China, Australia, Europe (specifically England, Sweden and the Netherlands) and the USA for this scoping study because they have policy drivers for integrating health in new development. We conducted this study internationally for two reasons: 1) the presence of international guidance documents and standards (e.g. WELL) suggest potential similarities across contexts; and 2) our research partner, Guy’s and St Thomas’ Charity (GSTC), requested knowledge about international best practice.

As healthy urban development is recognised as a relatively new area of professional practice (Marsh *et al*. 2020), we used purposive and snowball sampling to recruit participants. We used professional contacts and LinkedIn to identify participants in the targeted geographical areas. We corresponded with potential participants over email to understand their suitability for the study based on two inclusion criteria for participants: 1) they were either a built environment or public health professional and 2) they had experience of working on new developments which have integrated health and wellbeing. Sixty-two potential participants were invited to take part, and 31 accepted, a response rate of 50%. Within the sampling process we repeatedly assessed the balance of participants over different geographic areas. The supplementary material provides further details about our process for ethics, transcription, translation, and participant recruitment.

### Participants’ professional roles

Table 1 summarises the geographical distribution of participants, alongside their organisation type and profession. Participants worked primarily in the built environment (27/31). Their work was often domestic in their country of employment, though some worked internationally. In the supplementary material we provide data about participants sector of employment and demographic information.

### Analysis framework

We analysed the data in Nvivo qualitative data analysis software (QSR International Pty Ltd., version 12.6.0, 2019) using thematic analysis (Braun and Clarke 2006, Nowell *et al*. 2017). We used deductive and inductive coding (Fereday and Muir-Cochrane 2006). Our predefined codebook was based on our research questions and conceptual approach to the study (see supplementary material). Both researchers read all transcripts and we coded the data twice (once each, using the same Nvivo file) and inductively derived categories and codes were added in both stages. The data were grouped into overarching themes through an iterative process involving regular reflection between researchers regarding the interpretation of the data.

The interview guide and thematic analysis were informed by systems thinking (Meadows 2008) in recognition of the complexity of healthy built environments and the process of new development (Pineo *et al*. 2020). Although we were interested in the challenges and opportunities for implementing healthy development, these classifications can be overly reductionist and may miss the important interconnections that are visible through a systems thinking lens (Nilsen 2015). In the interviews we asked multiple questions to understand drivers, conflicts or complementary agendas, challenges and opportunities. We inductively coded under these topics as categories or codes and then looked for causal relations between codes (the final codebook is reported in the supplementary material). Rather than undertake a comparative analysis examining the differences across geographic conditions, we focused on exploring the similarties in experiences across contexts.

## Results

This section begins by setting the context for the findings with participants’ perceptions on the drivers of healthy development. Then we report the key role of developers through a general classification based on their respective goals from development, as described by participants. This is followed by a description of the three themes that emerged from the thematic analysis: managing risk, responsibility and economic constraints; pushing business-as-usual practices; and building knowledge and capacity.

### Drivers of healthy building

The drivers for integrating health as a more explicit objective in development projects were described as emerging from regulations, standards, client briefs, government initiatives and changing public awareness. Some participants noted a shift in wider conceptualisations of health in their society. A Chinese participant said that *‘people start to think healthy means healthy taste, healthy living, quality’*. A similar view was evident in London where a sustainability and engineering consultant said: *‘* … *it just feels like everyone is very aware of issues around, not just inside buildings, but air quality and stuff’*. There was a perception that the office sector in particular is shifting toward attention to health, wellbeing and productivity (described under ‘Evidence of added value’).

Health and sustainability were explicitly linked by most participants, both when prompted and unprompted. An American urban designer said *‘my take on healthy communities is an evolved version of sustainability’*. Participants in all settings described health and sustainability as two overlapping objectives that are *‘intertwined’, ‘almost the same’, ‘very complementary*’, and *‘a very natural pairing’*. However, some tensions were raised. An architect in China responded to our question about the relations between sustainability and health by exclaiming *‘You’ve just hit Pandora’s box’*, in relation to the energy costs of better indoor air quality. In summary, participants had a broad view of the drivers for health in development, covering regulation, client briefs, public awareness and the link to the sustainability agenda.

### Classification of outcome- and output-driven developers

Participants had common ways of explaining developer’s willingness, or lack thereof, to pursue health and wellbeing outcomes through design, from which we derived a general classification of developers. Perceived differences among developers were not solely attributed to their sector (e.g. private or public) and common descriptions persisted across interview contexts. Table 2 outlines our classification of organisations leading new development, with those who are more willing to take a healthy design approach described as *outcome-driven* and those who are not described as *output-driven*. Each of these general classifications is illustrated with participants’ quotes below. We adopted the terms *outcome* and *output* based on Weiss (1998). Outcomes describe longerterm goals and societal impacts (e.g. supporting local economies, sustainability and health). Outputs describe shorter-term goals and impacts (e.g. property sales values and ease of leasing property).

Outcome-driven developers were described as taking a *‘long-term’* interest in the development. Organisations that looked after their interests (i.e. assets and reputation) over time included real estate owner/occupiers, universities, housing associations, government-owned developers and ‘*legacy landowners’*. A project manager in London explained how the type of developer influenced what could be achieved on the project. Their client was an owner/occupier (called a Real Estate Investment Trust) so they were ‘*pushing against an open door’*. Usually developers would ‘*build and then sell it, having added value’* but their client was interesting in learning from occupants, resulting in ‘*the next building they develop, being improved by feedback from them, so it’s in their interests to make buildings better’*.

Another feature of *outcome-driven* developers is an organisational ethos, described as *‘values based’, ‘mission driven’* or *‘enlightened’*. These terms were sometimes used to characterise commercial developers. In one case, the investors’ values and dedication to the community were seen to strongly influence a largescale mixed-use project in the USA: *‘* … *the goals that they gave us [were] to create the most sustainable design you possibly can, but build houses that are affordable to a broad range of people* … *that is not generally the type of investor that developers get to work with* … ’.

In contrast, the output-driven description typically related to *‘commercial’* or *‘private’* developers who were primarily focused on the financial *‘bottom line’*, with little regard for the long-term impact of their projects. One quotation contrasts these two classifications clearly:

“ … the education sector tend to be long-term owner/occupiers which is quite an important consideration, I think, when you’ve got big, commercial developer landlords just building and flipping buildings. Arguably, they’re less interested in the longevity of the people that are in it” (sustainability consultant, London).

This short-term focus was perceived by some participants as a systemic failure rather than a vilification of certain property developers, as the same consultant explained: ‘ … *fair enough, they’re businessmen. That’s what happens when you’ve got a housebuilding industry and on large scale, that has been set up to make money* … ’. Notably, both outcome- and output-driven developers were focused on finances. There was a perception that outcome-driven developers *‘want everything’* (e.g. homes that support health, sustainability, etc.) and ‘*they’ll want to sell them for a good price’*, yet their outlook towards these goals means that health-promoting design is more likely to be integrated, within financial limits. The next three sections describe the themes that emerged through our thematic analysis.

### Managing risk, responsibility and economic constraints

There were financial, professional and reputational risks that influenced how health could be promoted at different stages of urban development. Many of these perceived risks rested with the developer. Participants noted that making claims that a project would be ‘healthy’ or would adhere to a particular healthy building standard introduced additional risks than would otherwise be present. These risks related to a perceived lack of control about the measurable or perceived health impacts of development, sometimes due to the performance gap between design and occupation.

#### Risk of failure

Developers are aware of the risk of not producing a healthy development, if they pledge to do so in preconstruction phases. In one case, an English housing association representative explained how negative outcomes on a particular project increased the organisation’s perception of risk associated with publicly aiming for healthy development:

“Health, I think because you don’t have direct control over it … (…) we get all ambitious about it and then we get part way through a process and we sort of go, ‘oh, we haven’t made the impact’ and that’s really quite hard and I think that’s quite a big risk … ”.

The difference between the intended and actual performance of a building or place (i.e. performance gap) was frequently described as a risk. Participants noted that healthy building standards (primarily WELL) require many verifications (e.g. air quality) when the building is occupied. This results in risks for design teams, developers, landlords and tenants, with no single party being in full control of the outcome. Such standards also require that information is shared between actors that was previously not transparent, again contributing to perceived risk. An American sustainability consultant explained difficulty finding a new office space that would meet WELL: *‘* … *there were many landlords that just told us, “we’re not doing that, we’re not going to monitor and share information, that’s none of your business.” There’s a lot of that attitude out in the market place* … ’. The landlord and tenant had to form a *‘partnership’* and agree to take on risk together; both parties were mutually dependent to achieve and maintain the certification status.

In other contexts, standards were seen to de-risk healthy building processes because the responsibility can be shifted to the standard itself (i.e. for both success or lack thereof) and because such standards require post-occupancy verification. An engineer in the Netherlands explained their national challenge with the performance gap stating that they ‘*never verified if those requirements were met and even if they did, they had no way to get the contractor to improve the building to meet those requirements’*. The benefit of the WELL standard was described as its explicit requirement for performance verification. This example also exposes that the responsibility for performance in a new building is not fully resolved and actors are likely to resist exposing failures.

#### Economic risks

A primary risk (perceived and actual) associated with healthy urban design relates to the cost of going beyond business-as-usual. Participants explained that significant perceived risk is introduced with the objective to achieve healthy development through increased, and largely unknown, costs associated with design team knowledge gaps, expensive materials or technical systems, certification, community participation, maintenance and more. Because health is seen as a relatively new design goal, these costs are currently high. Furthermore, there is a lack of data about whether healthy buildings can achieve a higher value for commercial developers.

Several interview participants noted that cost-related risks will continue to drop over time as the supply chain (and potentially the public) respond to the healthy building agenda. An architect in China explained how this occurred on the RESET healthy building standard. Certification was *‘very expensive’* initially and limited to *‘luxury projects up until 2012’*. Over time, some of the technical *‘solutions have been commercialised and prices have just plummeted’* resulting in expansion of the standard to different development types and locations. Notably, the economic constraints and risks associated with healthy development may be dampened for developers classed as outcome-driven, for example if they retain properties and measure their return on investment over many years.

#### Taking risks: pilot projects

A key opportunity to overcome the perceived risks and typical economic constraints of new development was through a *‘pilot’* or demonstration project. Such projects were used to explore innovative practices without necessarily promising success. If projects are shown to be successful over time and for different objectives (e.g. environmental performance or cost), they become a *‘template’* for further work.

A Seattle-based architect described pilot projects as a means to de-risk the process for the public sector and allow an innovative ‘*biologically based, waste water treatment system’* to be adopted. The project was not compliant with local regulations so ‘*we called it an “experimental system” which then provided cover for everybody to monitor extremely carefully, but to allow [it] to proceed and then prove itself* … ’. Pilot projects were described in other settings as a means to try new design approaches, demonstrate viability and build client demand. In a similar vein, two participants described the last recession as providing an opportunity for the project teams to try innovative approaches.

This section has described the perceived risks associated with healthy design and building practices for different actors. Challenges associated with increased costs and potential for failure were offset through the use of standards and pilot projects. However, standards created their own challenges by increasing transparency about building performance, thereby potentially increasing risk and responsibility for some parties.

### Pushing business-as-usual practices

There was a clear perception that not all developers were willing to consider health objectives in their projects. Participants in public sector roles noted that without supportive policies in place, *‘we beg, we plead’*, to negotiate for amenities and health-promoting design. To move beyond requirements in regulations and planning policy (described here as ‘business-asusual’ or ‘standard’ practice), participants described building a business case, advocacy, collaboration and early engagement on projects.

#### Evidence of added value

Developers were frequently described as needing to be convinced of the added value of healthy developments, using *‘data’* and *‘evidence’* about health improvements or financial benefits from other projects. Participants noted that this evidence was not always available and therefore building a *‘business case’* was difficult. An American sustainability consultant explained the challenge of convincing developers of integrating healthy design strategies, either for a specific standard (in this case it was WELL) or more broadly:

“The problem is the price point. No developer is going to pay … (…) we’re still arguing the value of LEED certification, even though we can easily demonstrate how it pays for itself. (…) With health and wellness, it’s a harder sell because we don’t know if these good ideas will truly make us healthier or not because there’s no real good way to measure it”.

Evidence was seen as important to drive the design process, particularly because health and wellbeing was a relatively new design objective and architects had been relying on ‘*intuitive’* knowledge. An American architect said *‘* … *I would say, we’re in the precautionary period, as opposed to having the data to prove that the things we think we’re doing actually are right* … .*’*. Similarly, an Australian architect said: *‘* … *we haven’t quite gotten to the point where we can put some data up and put some numbers against it and actually say to people, “this is a good thing because this, this and this*”’. These excerpts show design professionals’ interest in measuring the outcomes of their work, because the data about impact becomes part of the business case to inform future projects.

The kind of evidence that could be used to build a case for healthy design was not only from project monitoring, but also from health impact assessment. An American urban designer and planner noted that evidence about local health and built environment-related challenges helped their team to negotiate with the city for specific adjustments to local policy to support health outcomes in a large new community: *‘* … *by taking this health approach, what we were able to do was to have some data and evidence to build the case for why certain interventions were important’*.

There was an indication from some participants of a recent shift in developers’ conceptualisations of economic value, from short-term costs to long-term gains, thereby affecting how design teams can make a business case. A sustainability consultant in London said:

“It just fundamentally boils down to what is, in essence, a business case. It’s, ‘alright, we’re happy to spend the money, but what value does it deliver?’ I think there is … maybe not a macro shift, but certainly, more than a micro shift in … it certainly seems like people are starting to consider, more comprehensively, whole life cost, return on investments, pay back, all those sorts of things … ”.

This shift in attention to wider values was linked to other participants’ descriptions of changing demands from office space tenants in particular (tenants who are competing for highly educated and *‘talented’* staff). This has resulted in greater focus on the perceived quality of space and its actual impact on staff, including metrics such as stress and productivity.

#### Ways of working: advocacy and negotiation

Negotiation, *‘advocacy’* and *‘leadership’* were described as ways to mobilise actions towards healthy urbanism. Participants talked about bringing together stakeholders’ diverse agendas and goals, recognising the importance of the social aspects of healthy placemaking. One sustainability consultant explained that *‘* … *the barriers are not technical’* and instead *‘it’s all stakeholder oriented, it’s all political. … it involves compensation and money and ego and huge institutions and organisations that are not easy to penetrate or to change … ’*.

Participants spoke of discussing, persuading, negotiating, and influencing to bring forward specific healthy design measures. An American planner said *‘ … it’s all about telling a story in a vision that really does spark change’*. It was seen as valuable to have multiple parties from different sectors communicating the same message: ‘*it’s tough going if you’re the lone voice*’. Advocates or *‘champions’* were seen as important to set out and maintain the level of ambition, with one English housing association participant remarking that a project champion needed to *‘fight his corner’*. On one large-scale English development, a local GP (outside of the project team) was an *‘absolutely critical person’* in *‘justifying’* the case for healthy design measures and working across local stakeholders to move the project forward.

#### Practices of collaboration and communication

Collaboration and sharing knowledge between parties were key tools for those working within the field of healthy urban development. Collaboration supports information sharing, the generation of new insights, and the broadening of networks. Communicating a healthier living approach was seen as a *‘powerful’* message that *‘* … *helped us prioritise what strategies were hitting on multiple benefits or goals’*. Adopting healthy building standards helped an architect in Sweden to work across disciplines: *‘it actually knits together the people that are working with health and wellbeing and all of these experts because we’re having more profound discussions’*. In other cases, the goal of collaboration was to open up design and decision-making to a wider range of voices, which some participants reflected as being a difficult process.

An American urban designer described a university campus design project that sought to bring in students’ voices, particularly regarding race and other under-represented groups in the design process. The client wanted to know ‘*if there was racial bias in the design of their spaces’*. This was seen as a new concern: ‘*it really has been the first time an institution wanted to see how the school’s design … just didn’t create a sense of comfort or wellness for many students’*. A series of meetings were organised to speak with under-represented groups on the campus, including those related to race, sexual orientation, disability and military service. The design team was surprised by the findings. They thought that ‘*the older buildings on campus would be unwelcoming, they would appear too neo-classical or they would just look like white spaces, from euro-centric design bias’*. In fact, the meetings showed that in those spaces students ‘*felt cosy, they felt warm, they felt like they were designed for students’*. Instead it was the new buildings that were problematic; they *‘felt really cheap’* and there were *‘too many transparent spaces, not enough spaces to hide or feel comfortable’*. The designer said the students’ aversion to transparent spaces was about wanting to ‘*feel like it’s our own, cosy space’*. One student mentioned the risk of active shooters and the designer noted that ‘*the idea of safety and security is much higher’* than the design team had understood. This discussion highlighted the importance of participatory processes to raise under-represented voices in healthy design processes.

#### Early engagement

Early discussion of health-related objectives was important to ensure achievement of those goals within a development. Likewise, early engagement with end-users, typically over a client brief, was a strategy to understand their requirements and have time to respond. An Australian planner said *‘we were fortunate to be in the early stages with the client and so we were able to encourage him to include those features’*. In that case, the features related to landscaping, shade structures and solar panels. The developer was initially concerned about cost implications but the planners were able to frame that cost as having wider value: *‘they could use this as a marketing tool’*.

This section has described the multiple strategies that can be used to push for better practice than business-as-usual development. Design teams and consultants frequently had to make a business case for healthy design measures using evidence and data from monitoring, health impact assessment or scientific studies. Advocates helped to ensure that commitments were made and retained throughout a project. Collaboration and communication across project partners and sectors helped to make a business case, but could also produce challenges to the design process. Finally, early engagement in the design and planning process was important to influence the integration of health measures.

### Building knowledge and capacity

The final theme relates to the built environment sector’s current ability to integrate health into new development. There were conflicting views whereby some participants highlighted knowledge gaps and others focused on what is already known. Capacity refers to a broad set of factors, including the availability of non-toxic building materials, time to engage all actors and permission from decision-makers.

#### Lack of public awareness

Public awareness about buildings and health was viewed as low (although possibly increasing), across all regions. Participants noted that low public awareness reduced demand for healthy buildings, at least in the residential sector, but possibly more widely due to the potential political or market pressure that could result from public awareness. An English sustainability consultant said: *‘* … *why would an end user know about their indoor air quality or pollutants* … *I guess [developers are] betting on that not being on people’s agenda when they’re buying a new house* … *’*. Similarly, a Chinese sustainability and engineering consultant felt that low public awareness affected the business case for healthy development: *‘* … *a lot of building users they don’t know how many categories are related to their health* … *If they didn’t have awareness they would not seek for the office or residential with this kind of a good design.’* Some participants felt that organisations producing building standards should try to increase public knowledge to shift demand.

#### Knowledge among professionals

Despite recognising some gaps in the scientific evidence base about health and the built environment, participants generally said that there was enough knowledge to act now. However, not all participants reflected this view. For example, an American sustainability consultant noted that knowledge of toxins in building materials was low in the sector: *‘* … *you have to have a PhD in chemistry to understand what’s in those* … *’*. Furthermore, such knowledge requirements were constantly shifting *‘as one substance gets eliminated* … *then another substance takes its place* … *’*. A Swedish property developer explained knowledge gaps among real estate managers and others, citing a need to fix this through communication: *‘* … *you have to try to get it out, so all the consultants, the architects, the constructors, even the tenants, get the knowledge, so it’s a lot of hard work* … *’*.

Participants valued different forms of knowledge, not just evidence and research, but also relying on tacit knowledge from experience and *‘intuition’*. For example, an Australian architect described their personal reflections, *‘making sure that each step, each building that we do is better than the last one’*. To achieve this, the architect built knowledge from their experience over time by *‘writing these principles down and saying, “what is a non-negotiable in [one of our buildings]?” and learning from each one’*.

Potential knowledge gaps were downplayed by some participants, particularly when compared to financial barriers. An American sustainability consultant said: ‘*We can design and build healthy all day long, it’s not that difficult, if the client’s willing to do it and they want to spend the money, we have the expertise, it’s not rocket science*’. However, it was not only technical knowledge that was seen as necessary, but also knowledge on ways of engaging and encouraging participation in design, as described in the university campus project above.

#### Increasing capacity in the sector

Professional bodies and informal networks were described as key factors for sharing good practice and building capacity in the sector. A range of professionals were involved in knowledge production, validation, legitimisation and dissemination. A planner in Australia described how a small group of interested professionals and academics (in the Premier’s Council for Active Living) were drivers of the healthy placemaking agenda in Sydney and elsewhere in New South Wales. Through their ‘*communication’* and ‘*coordination’* they brought ‘*different government departments together’* to make things happen. They also used *‘demonstration projects’* for building capacity as they *‘educate people and then sharing the learnings and having it infiltrate across different skill sets* … *’*.

In summary, many participants emphasised that healthy buildings and development are an emerging field that provides opportunities for different forms of knowledge, innovative ways of learning, and new ways of sharing learning. Whilst some professionals identified specific knowledge gaps (e.g. toxins in building materials), others felt that the sector knows how to design healthy places, pinning finances as the key barrier to progress. Some participants highlighted the importance of gathering knowledge held by future building or neighbourhood occupants/residents to ensure that health is integrated into projects. Finally, informal and formal networks were described in several settings to fill gaps in professional knowledge, including through the use of demonstrator/pilot projects.

## Discussion

This exploratory study has contributed insights into built environment professionals’ experiences of implementing healthy urban development. We have provided a more nuanced understanding of developers’ roles in this process of change, exploring how their diverse goals and risks must be accounted for when attempting to persuade them to adopt healthy design measures. Our research has also highlighted specific strategies to push beyond business-as-usual practices, through: the creation of data about health impacts (and increased value) to build a business case; adopting pilot projects and healthy building standards; using champions within or outside the project team, engaging early in the design process; and increasing public awareness and professional knowledge through communication. In this section, we consider our findings in relation to existing research and theory. First we describe the strengths and limitations of our approach. Then we discuss potential mechanisms to manage developers’ risks, including networks and standards. We then consider the potential impact of Covid-19 on our findings related to public and professional awareness of healthy places. Finally, we consider the importance of reflective practice and diverse knowledge types from a multi-level learning perspective.

This study adopted semi-structured interviews to explore professionals’ experiences of integrating health into new development across six countries. The credibility of the study is reinforced by the confirmation of some of our findings in scholarly and professional literature. For example, supporting innovation in sustainable design and construction has been achieved through champions, pilot projects and early engagement in the design process (Mills and Glass 2009, Chang 2018, Martiskainen and Kivimaa 2018). We have provided several detailed accounts from the interviews, and highlighted commonalities across geographies, to increase the transferability of findings. However there are limitations with this study. Our purposive and snowball sampling approach is not a representative sample, although we believe this strategy was appropriate for the area of enquiry. We acknowledge that participant numbers per country are limited, thus it was not possible to look in depth at single areas or to conduct a comparative analysis. The international sample meant that the analysis did not include extensive reviews of local policy and market contexts, which limits the depth of analysis and interpretation. In countries where participants spoke English as a second language, understanding of questions and responses may have been reduced for both participants and researchers. The literature review in China, Sweden and the Netherlands was also limited by publications that were available in English (although we were able to translate some key Chinese literature). In using video-conferencing for interviews we were able to invite participants from a range of geographical regions, but a key drawback was the difficulty in building rapport and noticing non-verbal cues (Lo Iacono *et al*. 2016). Given the limitations of this exploratory research, we focused our analysis on commonalities across contexts and we draw out priority areas for further research below. We acknowledge that this research is a starting point in an under-researched area.

Our study revealed the key role of developers in the integration of health in developments. Developers’ financial and reputational risks must be understood and managed to achieve healthy development. Ways to manage developers’ risks were described through pilot projects and using healthy building standards. Managing their *perceptions* of risk may be more challenging and relates to making a business case that the value created through the development will offset the costs. Both ‘value’ and ‘cost’ are diversely interpreted by the stakeholders involved in healthy placemaking and our general classification of developers highlights how a business case could respond to some of these different perspectives. Carmona (2019) provides evidence that high-quality development creates value for health, social, economic and environmental outcomes. Yet, Henneberry *et al*. (2011) argue that market structures prevent developers from accounting for these wider economic costs and benefits in their financial viability calculations. The distributed value of high-quality developments, in terms of health benefits alone, does not offset the costs for developers, creating a real challenge for implementation (Pineo and Rydin 2018). There is an unresolved question as to whether professionals can ‘convince private sector developers’, as Carmichael *et al*. (2019) argue is needed, with arguments about wider value. This points to inherent power differentials between developers and other stakeholders, but also a need to pragmatically evaluate the mechanisms that could change the viability equation for developers.

New incentives in the form of voluntary healthy building standards may shift the market, yet our research indicates that other incentives may be needed. Rydin (2010) describes incentives as mechanisms to change power dynamics and ‘alter the underlying frameworks setting the costs and benefits of decision making’ (p.58), including taxation, subsidies, transfer of landownership, and collaborative action through networks and partnerships. Participants did not describe examples of such financial incentives, yet we are aware of some examples, such as Fannie Mae’s Healthy Housing Rewards programme (Center for Active Design n.d.). Similar approaches could be identified and explored to understand their value to developers. Interview participants did describe both informal and formal networks in Australia, England and the USA that helped build capacity for healthy urban development. The existing literature on planning healthy built environments supports the importance of building cross-sectoral relationships to support collaboration and knowledge sharing over time (Carmichael *et al*. 2012, 2019, Lowe *et al*. 2018, Pineo *et al*. 2020). A recommendation for practice would be to strengthen these networks. The potential for further taxation, subsidies or incentives for healthy building is an area for further exploration in practice and research.

There was a perception that no matter how strong a business case can be made, some developers will not produce healthy development unless it is a legal requirement. It is unclear whether the Covid-19 pandemic (which occurred after our data collection) could shift the political acceptability of legal requirements for healthy development. The pandemic could have multiple effects on the healthy building agenda. It may shift focus to infectious disease prevention, which could have unintended consequences in excess energy consumption in building ventilation systems, ultimately harming humans through planetary and ecosystem health systems (Pineo 2020). Covid-19 may increase public awareness about health and place, yet this requires further exploration. Interview participants called for more evidence that ties specific design interventions to health and wellbeing outcomes; however, it is unclear which parts of the evidence base need strengthening. Perhaps the greater challenge (that was raised by participants) is in communicating the evidence to increase general awareness, shifting the incentives for action. This is not a likely task for the research community alone, but perhaps one that can be taken up with diverse professional bodies using new communication techniques such as the ‘evidence-based framing strategies’ produced by Moyer *et al*. (2019).

Finally, we note that in practicing healthy placemaking, evaluative and critical reflection is an instrument for progress in order to build capacity and knowledge in the sector. Healthy development is an iterative process in which participants attempt to solve a problem, create a solution, critique that solution, and then take forward that knowledge in future practice. The interviews revealed plural and varied ways of learning, that seemed to respond to the ‘knowledge gap’, resulting in reflective practice. Models of multi-level, multi-loop and double-loop learning may describe this practice, drawing on systems thinking (Argyris and Schön 1974, Meadows 2008). In these models of learning, a person draws upon information in their environment (feedback) to modify their mental model of cause and effect relations in a particular system, and they use that altered model to shift decision-making in light of experience. Collaborative and shared knowledge can also be used to achieve such reflective practice (Diduck 2010). Gathering diverse types of knowledge is a recognised strategy to deal with the complexity and contested nature of development (Innes and Booher 2010) and the complexity of urban health challenges (Gatzweiler *et al*. 2018). Interaction with future building and neighbourhood occupants was not a widely discussed way to gather knowledge in the interviews, and this could be a recommendation for further improvement in practice.

## Conclusion

This exploratory study has contributed insights into potential approaches for integrating health into new development and priority areas for further research. The following conclusions focus on next steps for research and practice:

- Research about creating healthy places has primarily focused on planning policy, yet other stages of urban development are key to successfully negotiate and integrate health objectives. Future studies should explore implementation through the motivations and capabilities of different actors (particularly developers) in specific policy contexts and the role of development financing.
- Further research in specific policy contexts could explore the potential for financial or other incentives to improve healthy development and stakeholders reaction to different approaches. This relates to our finding that developing a business case is an important step to convince developers to incorporate healthy design measures, because the financial argument depends on local policy requirements, land values and other context-specific factors.
- The potential to build knowledge and capacity in the sector is key. There are similarities between the sustainable and healthy property agendas, not least because participants saw their respective goals as overlapping, but also because both agendas are seen as trends that require new knowledge, technology and ways of working. It may be helpful to increase sharing of knowledge and lessons for successful implementation of sustainability and health objectives, including through Environmental, Social, and Corporate Governance (ESG) processes.
- In relation to building knowledge and capacity within professional communities, we found evidence of evaluative and reflective practices, wider than building performance evaluation, that could be further explored and exploited to integrate health.
- There is a need to understand why professionals do not feel that the existing evidence base supports their design decision-making, and whether monitoring in specific developments could overcome this challenge.

## Supplementary Material

Table 1. Interview participants (n = 31) by country, organisation and profession

Table 2. Characteristics of outcome- and output-driven developers

## Figures and Tables

**Table 1:** Interview participants (n = 31) by country, organisation and profession.

Geographic area (participants)	Organisation type	Profession	No. of participants
Australia (6)	Planning commission	Planning	1
	Planning consultancy	Planning	1
	Architecture and design practice	Architecture	1
	Public health department	Public health	3
China (7)	Engineering consultancy	Sustainability and engineering	2
	Engineering consultancy	Landscape Architecture	1
	Architecture and design practice	Urban design and planning	2
	Building standard organisation	Project management	1
	Building standard organisation	Architecture	1
England (7)	Engineering consultancy	Sustainability and engineering	1
	Architecture and design practice	Project management	1
	Architecture and design practice	Sustainability	3
	Housing association	Research	1
	Public health department	Public Health	1
Netherlands (1)	Engineering consultancy	Indoor Environmental Engineering	1
Sweden (2)	Architecture and design practice	Architecture	1
	Real estate developer	Property Development	1
USA (8)	Engineering consultancy	Sustainability	1
	Architecture and design practice	Urban design (and planning)	2
	Architecture and design practice	Sustainability	1
	Architecture and design practice	Architecture	1
	Building permitting department	Planning	1
	Planning department	Planning	1
	Building standard organisation	Urban design and planning	1

**Table 2:** Characteristics of outcome- and output-driven developers.

Outcome-driven developers	Output-driven developers
Longer-term goals and interests	Shorter-term goals and interests
Broader, interconnected impacts	Narrower, focused impacts
Focus on finances	Focus on finances
Interests in societal impacts e.g. supporting local economies, sustainability and health	Interests in economic impacts as property sales value, ease of leasing property
Go beyond regulations	Meet regulations
Pilot, experiment	Follow known practice

## Data Availability

Due to the nature of this research, participants of this study did not agree for their data to be shared publicly, so supporting data are not available.
